# 16S rDNA sequencing and metadata of Dutch dental unit water

**DOI:** 10.1016/j.dib.2021.107221

**Published:** 2021-06-12

**Authors:** Michel A. Hoogenkamp, Bernd W. Brandt, Alexa M.G.A. Laheij, Johannes J. de Soet, Wim Crielaard

**Affiliations:** Department of Preventive Dentistry, Academic Centre for Dentistry Amsterdam, University of Amsterdam and Vrije Universiteit Amsterdam, Amsterdam, The Netherlands

**Keywords:** Dental unit water, 16S rDNA sequencing, Heterotrophic plate counts, *Legionella*, Amoeba, Bacteria, Fungi

## Abstract

Dental practices were approached to fill out a questionnaire on the infection control protocols in use to control biofilm growth in the dental unit and to send two types of water sample. Sampling of the dental units had to be performed prior to any infection control measures and on the second day of operation, to avoid residual effects of biofilm disinfection protocols performed in the weekend. Instructions were given on how to sample the units. Only samples, accompanied with a completed questionnaire and returned within two days by regular mail, were analysed. Samples were processed for heterotrophic plate counts, 16S (V4) rDNA microbiome sequencing and q-PCR for the concentration of bacterial 16S rDNA, fungal 18S rDNA, *Legionella* spp. and the presence of amoeba. The files contain the metadata needed to interpret and analyse the microbiome data. This dataset can be used by other scientists, members of infection control units, (trainee) bioinformaticians and policy makers. This dataset can provide leads to further unexplored parameters which could influence the microbial ecology of the dental unit.

## Specifications Table

**Table:** 

Subject	Immunology and Microbiology Microbiology
Specific subject area	Dentistry and public health
Type of data	Table
How data were acquired	• Heterotrophic plate counts were acquired by culturing on R2A agar (BD, Sparks, IL, USA) • Q-PCR data was acquired using Lightcycler 480 II hardware and software (Roche, Almere, The Netherlands) • 16S rDNA sequencing data was acquired using Illumina Miseq Technology, with V3 chemistry, 2×251bp and Miseq Control Software (MCS v3.0) Software. (Illumina, Cambridge, UK) • Behavioural data on infection control measures were collected using a questionnaire (see Suppl. file Questionnaire)
Data format	Illumina MiSeq sequencing reads Metadata
Parameters for data collection	Sampling of the dental units had to be performed prior to any infection control measures and on the second day of operation, to avoid residual effects of biofilm disinfection protocols performed in the weekend. To avoid external contamination of the samples, instructions were given on how to sample the dental units. Only samples, accompanied with a completed questionnaire and returned within two days by regular mail, were analysed.
Description of data collection	Dental practices were approached to participate and fill out a questionnaire on their infection control measures. Additionally, two types of water samples were sent. Samples were processed for HPC counts, 16S V4 rDNA sequencing and q-PCR for the concentration of bacterial 16S rDNA, fungal 18S rDNA, *Legionella* spp. and the presence of amoeba.
Data source location	Academic Centre for Dentistry Amsterdam Amsterdam, The Netherlands
Data accessibility	Microbiome and metadata Repository name: NCBI BioProject database Data identification number: PRJNA690093 Direct URL to data: https://www.ncbi.nlm.nih.gov/bioproject/PRJNA690093
Related research article	Michel A. Hoogenkamp, Bernd W. Brandt, Alexa M.G.A. Laheij, Johannes J. de Soet, Wim Crielaard Title: The microbiological load and microbiome of the Dutch dental unit; ‘please, hold your breath’ Journal: Water Research DOI: 10.1016/j.watres.2021.117205

## Value of the Data

• The data contains information of the concentration of bacterial 16S rDNA, fungal 18S rDNA, *Legionella* and the presence of amoeba in dental unit water and the external factors influencing this ecology.
• Scientists, members of infection control units, (trainee) bioinformaticians or policy makers can use this data.
• Further analysis of this data could improve infection control measures within the dental health setting which can be beneficial to the health of immune compromised patients visiting the dental office.
• This data can provide leads to further unexplored parameters influencing the microbial ecology of the dental unit. The links between fungal, bacterial and amoebal DNA can further be explored.
• The following relationships were not analysed in detail in the related research article:

Relationship between the microbiome and the following parameter:

■ PCGroup
■ Water supplier
■ Unit brand
■ Unit Age Group
■ Water Type
■ Connection
■ Disruption

## 1 Data Description

NCBI BioProject: PRJNA690093: This accession contains the Illumina MiSeq paired-end sequencing reads sequencing data reads and meta-data (MIMARKS) of the sequenced data.

Metadata.xls: This file contains the metadata used for statistical analysis on the entire sample set and also include the metadata from samples which could not be sequenced due to low DNA yield. This dataset can be linked to the microbiome samples using the sample ID. Data of each sampled dental unit was obtained from the questionnaire and microbiological analysis performed during the original study. Data groups are specified and explained in tab: Coding Columns

## 2 Experimental Design, Materials and Methods

### 2.1 Selection of the dental practices

A large group of dentist in The Netherlands are members of the Royal Dutch Dental Society (KNMT) and provided permission to be approached for scientific studies. From this group, a randomly drawn sample was selected to be approached for this study specifically (*n* = 921). Participants did not receive any compensation and participated on a voluntary basis. Contact details provided by the participants were also used to ask additional questions and to complete possible omissions in the questionnaire. When the participants wanted to receive the outcome of their bacterial counts (HPC), this was dealt with separately by a dentist, involved with this study, but not involved with data analyses. Upon arrival in the laboratory the questionnaire and samples were coded and blinded to the operator. The moment all details of the study were received and the HPC data were communicated, the names and addresses of the dentists were deleted completely, as agreed with the KNMT.

Sample collection:

Practices were sent the following items:

1. an invitational letter describing the purpose of the study
2. a questionnaire on the water management protocols in use (See Suppl. File Questionnaire)
3. a sampling kit, consisting out of:
  a. blue return envelope (Polymed, Daklapack Europe B.V., Lelystad, The Netherlands)
  b. one 15 ml sterile tube (Sarstedt, Nümbrecht, Germany, red cap)
  c. two sterile 30 ml tubes (Sarstedt, white cap)
  d. two postal stamps.
4. a sampling protocol (See Suppl. File Sampling instructions)

Participants were asked to fill out the questionnaire as complete as possible and were given the choice to be updated on the water quality of their dental unit. To avoid external contamination, dental practices were given clear instructions on how to disinfect the outside of the unit and on how to sample the unit (See Suppl. File. Sampling instructions.doc).

In short, preceding any flushing of the unit and prior to any infection control measures, a 50 ml effluent sample (proxy biofilm sample of the biofilm in its relaxed state (relaxed biofilm sample, RBS), was taken a-septically from the air rotor handpiece. Subsequently, the units were flushed for 30 s and a second 10 ml effluent sample was taken.

Samples were returned by regular mail and were processed immediately. Only samples which were processed within 48 samples were considered for analysis [1]. Samples were collected over a 5 month period from February till June 2019. No replicate samples from the same dental unit were collected. Quality assurance of the effect of transit time on HPC has been described in the Water Research paper [1].

Questionnaire:

Behavioural data on infection control regimens was collected using a questionnaire (see Suppl. File Questionnaire.doc).

Sample processing:

*Heterotrophic plate counts*

Heterotrophic plate counts were determined in the 10 ml effluent sample. These samples were vortexed and 100 µl was used to prepare ten-fold serial dilutions in sterile MilliQ. These dilutions were then plated, in duplicate, onto R2A agar (BD, Sparks, IL, USA) using an Eddyjet Spiral plater (IUL, Barcelona, Spain). All plates were incubated under aerobic conditions at 23 °C for 7 days [2].

As a negative control, sterile MilliQ, used to dilute the samples was also plated in duplicate to exclude external contamination due to the dilution and plating process.

*DNA extraction*

Samples were processed for DNA analysis by filtering 50 ml of the RBS sample using a Swinnex filter holder containing a nitrocellulose membrane (both Millipore, Ireland, ø25 mm, 0.2 µm pore size). Filters were transferred to a 5 ml Eppendorf tube and stored at −80 °C. DNA was subsequently extracted from the entire filter using a PowerBiofilm Kit (Qiagen, Roermond, The Netherlands [1]. DNA extraction kit blanks and filter blanks (unused filter from the filter holder) were incorporated and used to validate whether external contamination was introduced during the sample processing and DNA extraction.

*Microbiome analysis*

The DNA concentration was quantified using a q-PCR specific for 16S rDNA and the *V4* hypervariable region of the 16S rRNA gene was amplified [3]. The amplicons were equimolarly pooled and paired-end reads of 251 bp were generated using the Illumina MiSeq platform and Illumina MiSeq reagent kit V3. The sequence data was processed and a taxonomic name was assigned to the representative (most abundant) sequence of the operational taxonomic unit (OTU), based on the SILVA ribosomal RNA database, version 128 [2].

Negative PCR controls (molecular grade water) were incorporated in the microbiome analysis to validate whether cross-contamination had taken place. As a positive control, mock-samples were incorporated (BEI resources.org).

*Q-PCR analysis*

Q-PCR was performed using Lightcycler technology and chemistry (Roche, Almere, The Netherlands). All RBS samples were analysed, in duplicate, using molecular grade water (Thermo Scientific) as a negative control.

*The presence of Legionella spp., L. pneumophila and L. pneumophila SG1*

The presence of *Legionella* was determined, using 10 µl template DNA, according to Collins et al. [4] Calibration curves were constructed using *L. pneumophila* DSM 7513 DNA (DSMZ, Braunsweig, Germany) with a detection limit of 10 Genomic unit numbers (GU) per reaction (2000 GU l^−1^) for *Legionella* spp. and 1 GU for *L. pneumophila. Legionella* positive samples were verified by melting point analysis and the number of GU was calculated based on the inclusion of certified genome unit numbers in the q-PCR (LGC standards, Wesel, Germany) [1].

*The presence of fungi*

The fungal 18S rDNA concentration was determined using 5 µl template DNA, according to Wagner et al. [5]. Calibration curves were constructed using *C. albicans* SC5314 DNA

*The presence of amoeba*

To detect the presence of the amoeba *Acanthamoeba* and *Naegleria*, a conventional PCR was used [6]. Presence of both amoebal species was determined by DNA gel electrophoresis (60 min, 100 V, 3% Tris-Acetate-EDTA agarose (Fisher Scientific, Landsmeer, The Netherlands) on the PCR samples. The fragment length was determined using a 50 bp Generuler (Fisher Scientific)

The presence of *Hartmannella* was detected using 5 µl DNA template q-PCR specific for *H.vermiformis* [1], [7].

## Ethics Statement

No Medical Ethical approval was needed for this study. However, this study was approved by the Ethical Committee of the Academic Centre of Dentistry (ACTA, Amsterdam, The Netherlands) with special notice for Dutch privacy guidelines, under reference 201904SOET.

## CRediT Author Statement

**M.A. Hoogenkamp:** Conceptualization, Analysis, Data Curation, Writing original draft, Writing Review & editing; **B.W. Brandt:** Analysis, Software, Data Curation, Writing Review & editing; **J.J. de Soet:** Conceptualization, Writing Review & editing, Supervision; **A.M.G.A. Laheij:** Conceptualization, Writing Review & editing; **W. Crielaard:** Conceptualization, Writing Review & editing.

## Declaration of Competing Interest

The authors declare that they have no known competing financial interests or personal relationships which have, or could be perceived to have, influenced the work reported in this article.

## Supplementary materials

Image, application 1

Image, application 2

Image, application 3
