# Supplementary material for: 16S rDNA sequencing and metadata of Dutch dental unit water
Source: Data Brief. 2021 Jun 12;37:107221. doi: 10.1016/j.dib.2021.107221 (PMC8213887; doi:10.1016/j.dib.2021.107221)
Supplement: Supplementary file 2 [file mmc2.docx]

This is the questionnaire belonging to the unit you are sampling at this moment. Try to answer the open questions as complete as possible.

If you would like to receive an indication of your unit’s water quality you can write down you e-mail address below.

This mail address will only be used for this purpose and the end results can’t be traced back to individual practices

………………………………………………………......................................……

Date of sampling: ......-......-……

Time of sampling: _ _ : _ _

Postal code (Only first 3 digits to identify your drinking water supplier: _ _ _

1 What is the Brand, type number and year of purchase of your dental unit?

2 How often is maintenance performed on your dental unit?

3 Is the dental unit attached directly to the drinking water mains? ○ Yes ○ No

4 Are you using an external water reservoir to feed water to your 3-way syringe and rotational hand instruments (A bottle which has to be fed with water)? ○ Yes ○ No

1. With what type of water do you fill this external reservoir?

○ Tap water ○ Distilled water ○ Sterile water ○ Mineral water

○ Filtered water ○ Other, being:

1. Did you experience any disruption in your drinking water supply in the last year?

○ Yes ○ No ○ Don’t know

7 Do you flush your dental unit at the start of the day and if so, how long?

8 Do you use a disinfectant to treat the waterlines of your dental unit? ○ Yes ○ No

9 If so, how do you disinfect the waterlines of your unit?

○ Chemically (e.g Oxygenal or Alpron) ○ Thermal ○ Using a filter

○ Flushing with water ○ Other, being:

10 Do you add a daily disinfectant to your unit water (either automatically or manually to the external reservoir? ○ Yes ○ No

11 If so, which disinfectant do you use and how is this administered (automatically or by hand)?

12 Do you perform a biofilm removal protocol (Shock dose) on this unit?

○ Yes, which day?.................. ○ No

13 If yes, how often do you perform this shock dose, which disinfectant do you use and how long do you treat your waterlines?

14 Does your practice have a watermanagement plan to ensure the waterquality of your unit? ○ Yes ○ No

15 Who executes the water management plan?

○ Dentist ○ Assistant ○ Other, being:

If you have any additional information you can write this down in the box below.
