# Supplementary material for: 16S rDNA sequencing and metadata of Dutch dental unit water
Source: Data Brief. 2021 Jun 12;37:107221. doi: 10.1016/j.dib.2021.107221 (PMC8213887; doi:10.1016/j.dib.2021.107221)
Supplement: Supplementary file 3 [file mmc3.docx]

**Protocol Sampling dental units**

To obtain an, as accurate as possible, estimation of the microbial flora in your dental unit water it is of importance to follow the protocol below.

**Prior to sampling**

- Do NOT flush your dental unit prior to sampling.
- Take the sample on the second day of operation, preferably the Tuesday or the Wednesday.
- Only use the air rotor connection, without attaching the handpiece itself to avoid intrinsic contamination of the handpiece itself.
- Do NOT take a sample immediately after the application of a Shockdose/Biofilmremoval treatment.
- Take all items out of the blue return envelope (Polymed, Daklapack Europe B.V., Lelystad, The Netherlands). The envelope should contain: one questionnaire, one 15 ml sterile tube (Sarstedt, Nümbrecht, Germany red cap), two sterile 30 ml tubes (Sarstedt, white cap), two postal stamps.
- Only use the tubes supplied in the sampling kit.

**Procedure**

- Fill out the questionnaire as accurate and complete as possible
- Remove the air rotor handpiece.
- Put on clean gloves.
- Use 70-80 ethanol to disinfect the outside of the hose with the air rotor connector.
- Take the two 30 ml tubes (with the white cap).
- Take off the caps and place them on a clean surface (inside of the cap facing upwards).
- Fill both tubes with 30 ml water from the air rotor hose.
- Close the tubes.
- Flush the air rotor handpiece for 30 seconds.
- Take the 15 ml tube (with the red cap)
- Take off the cap and place them on a clean surface (inside of the cap facing upwards)
- Fill the tube with 10 ml water from the air rotor hose.
- Close the tube.
- Place all three tubes and the questionnaire in the blue envelope.
- Place the two stamps on the right top corner of the blue envelope.
- Store the envelope in the fridge till postage.
- Post the envelope the same day **BEFORE** 18:00 to ensure timely postal transit.
